# Ab Initio Molecular Dynamics Simulations of Phosphocholine Interactions with a Calcium Oxalate Dihydrate (110) Surface

**DOI:** 10.1021/acs.cgd.4c01032

**Published:** 2024-09-18

**Authors:** Rhiannon Morris, Helen F. Chappell, Andrew J. Scott, Antonia Borissova, James Smith

**Affiliations:** † School of Food Science and Nutrition, University of Leeds, Woodhouse Lane, Leeds LS2 9JT, U.K.; ‡ School of Chemical and Process Engineering, University of Leeds, Woodhouse Lane, Leeds LS2 9JT, U.K.

## Abstract

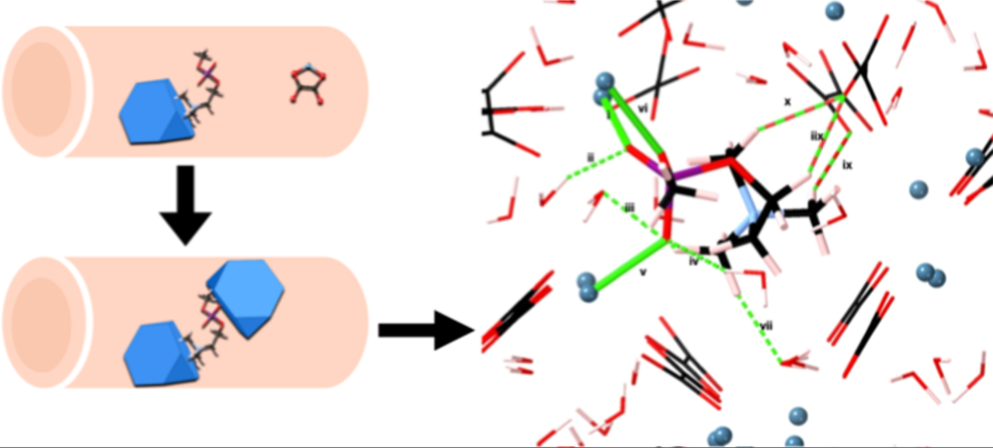

We use *ab initio* modeling (CASTEP) to help elucidate the crystallization phenomena and chemistry behind kidney stone composition and formation. To explore the stone formation process, we have constructed a surface model of calcium oxalate dihydrate—the mineral most commonly found in patients with hypercalciuria and modeled stone growth, by simulating further calcium oxalate adsorption onto the surface (−7.446 eV, −0.065 eV/atom). Furthermore, urine analysis of kidney stone patients has previously revealed that their urine contains higher concentrations of phospholipids compared to healthy individuals. Therefore, to investigate the interactions between urinary macromolecules and the growing crystal surfaces at an atomic level, we have performed *ab initio* molecular dynamics simulations of phosphocholine adsorption on calcium oxalate surfaces. We have shown that the phosphocholine headgroups become entrapped within the growing crystal and the lowest energy structures (−18.008 eV, −0.0396 eV/atom) are those where the calcium oxalate dihydrate surfaces have become disrupted, with reorganization of their crystallographic structure. Urinary calculi (kidney stones) are a common ailment affecting around 10% of the world’s population and resulting in nearly 90,000 finished consultant episodes (FCE) each year in the United Kingdom [Hospital Episode Statistics, Admitted Patient Care—England, 2011–12 NHS Digital, 2021–2022. https://digital.nhs.uk/data-and-information/publications/statistical/hospital-admitted-patient-care-activity/hospital-episode-statistics-admitted-patient-care-england-2011-12].

## Abstract 2

Using *ab initio* molecular dynamics simulations, we show that phosphocholine can encourage growth of kidney stones by binding crystallites together and providing a nucleation point for stone growth within the kidney tubules.

## Introduction

Today, kidney stones (urinary calculi) affect around 10% of the global population with a higher prevalence in both the USA (15%) and the Middle East (25%).^2^ Urinary calculi are frequently a recurrent problem, with an estimated 40–50% chance of a secondary episode,^3^ and an estimated cost to the USA of $4.5 billion annually in their treatment.^4^ In the UK context, between April 2021 and March 2022, there were 88,385 Finished Consultant Episodes recorded in England, for kidney stone hospital admissions.^1^

The mineral composition of stones varies, with calcium oxalate or calcium phosphate accounting for around 80% of all kidney stones.^5^ Calcium oxalate (CaOx) stones are by far the most common, forming approximately 70% of all identified stones.^6^ Calcium oxalate has three crystalline forms: calcium oxalate monohydrate (COM), calcium oxalate dihydrate (COD), and calcium oxalate trihydrate, although the latter polymorph is rare.^7^ In patients with hypercalciuria, stones are usually composed of pure COD or a mixture of COM, COD, and phosphate, but there is nevertheless a wide variation.^8^

Kidney stones are, on average, composed of 97% mineral, with the remaining 3% being an organic matrix made up of macromolecules such as lipids and proteins.^9^ There have been investigations into the interactions of calcium oxalate dihydrate with aspartic acid peptides,^10^ poly(acrylic acid),^11^ and glutamic acid,^12^ and in each of these cases crystal habit changes were observed. For example, phosphorylated peptides of osteopontin proved inhibitory in the development of the COD crystal structure, resulting in the formation of rosette-like crystal aggregations (spherulites) and preferential binding to the (110) face.^10^ Additionally, Iwata et al.^13^ demonstrated, using scanning electron microscopy (SEM) imaging, that separate, distinct layers of COM and COD are found within the kidney stones, with organic matrix filling the spaces between interlocking COD crystals. A similar observation was made by Sivaguru et al.^14^ who suggested a complex process of COM and COD formation, dissolution, and remodeling that results in “entombed” biomacromolecules present in distinct organic layers.

A computational study from Debroise et al.^15^ suggested that the (110) surface of COD is the lowest energy surface, meaning it is the most stable surface, and is therefore more susceptible to adsorption of organic molecules. Indeed, the COD (110) surface has been observed experimentally as the strongest binding surface for both polyaspartic acid and osteopontin.^16^ While the processes involved remain unknown, the control of mineral growth *in situ* is clearly modulated by the organic matrix, suggesting that the minor bioorganic components are essential in the promotion of crystallization.^17^ Notably, compared to healthy individuals, a number of phospholipids, including phosphocholine, phosphatidylinositol, and phosphatidylethanolamine, have been observed at elevated concentrations in 24 h urine collections of stone sufferers.^18^ This association was particularly notable in calcium oxalate stone patients, whose phospholipid excretion was measured at 0.718 mg/24 h, which compared to 0.402 mg/24 h in uric acid stone patients, and 0.315 mg/24 h in healthy individuals.^18^ In the same study, the lipid content of the stone organic matrix was found to be 80% lipids in calcium oxalate stones (20% protein), 67% lipids (33% protein) in calcium phosphate stones, and just 25% lipids (75% protein) in uric acid stones.^18^ Biological mineral nucleation associated with lipids has certainly been observed in other pathological calcifications, for example, an organic core composed of phospholipids has been observed in submandibular salivary gland sialoliths and parotid gland sialoliths, with phosphocholine being the predominant phospholipid identified in the latter.^19,20^ Given the composition of the lipid component identified in these earlier studies, it is strongly suggested that epithelial cell membrane debris from dead or damaged cells (rather than, say, bacterial cell membranes) is the source of these organic components.^19,20^

Broadly, the outer leaf of epithelial cell membranes consists mainly of phosphocholine and sphingomyelin.^21^ To explore the chemistry and thermodynamic stability of phosphocholine as the nucleation point for kidney stones on the outer cell membrane of the kidney epithelial lumen, we simulated the adsorption of a phosphocholine headgroup onto the COD (110) mineral surface. The calculation was designed to determine the potential of this membrane macromolecule to be a stone nucleation point. In addition, however, the calculations help to establish whether free phospholipids, as detected in the urine of stone sufferers,^18^ would be able to bind to free crystallites, to either promote or inhibit stone growth away from the cell membranes. We explored the dynamics of unconstrained stone formation further by simulating the interaction of phosphocholine between two crystallite surfaces, allowing us to understand whether the phospholipid may have a role in crystallite accumulation during stone growth. We compare these simulations to crystal growth unmediated by organic molecules by simulating the adsorption of calcium oxalate directly to the surface.

Density functional theory (DFT) calculations, which accurately calculate thermodynamically stable electronic structures, inform us of the chemistry occurring between the inorganic and organic materials at the atomistic level, pinpointing the fundamental characteristics that bind individual crystals together in the kidney stone. Understanding stone formation and determining the causes of further growth are crucial to the development of kidney stone research.

## Methods

### Mineral Structure

During the past 40 years, the structure of COD has been refined utilizing advances in experimental techniques.^22−26^ The unit cell chosen as the starting structure for this work came from Izatulina et al.,^25^ who reported the formula as Ca(C_2_O_4_)·(2 + *x*)H_2_O, where *x* is defined as 0.37 and the space group *I*4/*m*. The structure was selected due to its being obtained from X-ray diffraction studies on 17 kidney stones from St. Petersburg citizens aged 24–65 years.^25^ The main difference between the 17 alternative structures in this kidney stone study is the amount of zeolitic water present in each unit cell. The zeolitic water has four alternative positions within the unit cell and can, on average, occupy one position at any given time (Supporting Information, Figure S1); this water is contained in the water channels and is highly mobile.^25^

To assess these positions, the unit cell^25^ geometry was optimized with a water molecule at each position in turn, and the lattice parameters of optimized and experimental unit cells were compared, along with the energy. The position at which the lattice parameters deviated least from experimental results^25^ was taken as the most accurate representation of the experimental data, as the energy change between structures was negligible (<0.001 eV). The optimum position for the zeolitic water was located at the purple sphere in Supporting Information, Figure S1. The unit cell volume change at this position was the lowest, with a 5.85% increase in unit cell volume compared to the experimental starting structure. Once the geometry was optimized, our work was in good agreement with the synthetic phase-pure COD unit cell reported by Izatulina et al.,^26^ the volume deviating by just 0.09% from their reported experimental volume. Using this unit cell, a (110) surface was cut, which encompassed two unit cells in the *y*-direction and with a depth (*z*-direction) of one unit cell.

### Computational Methods

Geometry optimizations used the DFT plane-wave code CASTEP,^27^ employing on-the-fly pseudopotentials and a plane-wave basis set with a cutoff energy of 900 eV. The Perdew–Burke–Ernzerhof^28^ functional with the generalized gradient approximation (PBE-GGA) was used to describe the exchange correlation. For surface calculations, the Tkatchenko–Scheffler (TS) dispersion correction^29^ was employed to describe the van der Waals interactions. Geometry optimizations were obtained by minimizing the total energy using a conjugated gradient algorithm within 10^–4^ eV. The forces on each ion were considered converged when less than 0.03 eV/Å, the maximum displacement within 0.001 Å, and with the maximum stress within 0.05 GPa. A Monkhorst–Pack^30^*k*-point grid of 2 × 2 × 2 was used for the COD unit cell, the *k*-points having been converged to within 0.01 eV. The unit cell lattice parameters were allowed complete freedom in a *P*1 space group, for full geometry optimization.

The vacuum gap for the surface was converged until the total energy varied by less than 0.1 eV, resulting in a vacuum gap of 12 Å being selected. The *k*-points were similarly converged until the total energy varied by less than 0.01 eV, equating to a *k*-point spacing of 0.06 Å^–1^. Geometry optimizations were obtained by minimizing the total energy using the same convergence criteria as outlined above for the unit cells. The lattice parameters were fixed to ensure the vacuum stayed at the converged depth. The phosphocholine headgroup (PC) was geometry-optimized, in a convergence-tested simulation box three times its size (30 × 30 × 30 Å), using a *k*-point Monkhorst–Pack grid of 1 × 1 × 1, with the molecule at the center of the simulation box.^30^

Adsorption energies (eq 1) were calculated to compare binding preference of both conformations of the same adsorbate and also between adsorbates. The lower the adsorption energy, the more favorable the interaction.
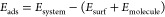
1where *E*_ads_ is the adsorption energy, *E*_system_ is the final energy of the geometry-optimized surface adsorption model, *E*_surf_ is the energy of the surface alone, and *E*_molecule_ is the final energy of the geometry-optimized molecule, calculated with a plane-wave cutoff energy of 900 eV.

Surface energy of the systems was calculated using eq 2.
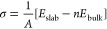
2where σ is the surface energy, *E*_slab_ is the total energy of the slab model of the surface, *E*_bulk_ is the energy of one formula unit of the bulk material, *n* is the number of until cells in the slab model, and *A* is the total area of the surfaces (top and bottom) in the model.

In this work, we will refer to Mulliken population analysis, which divides the electron density across the nuclei in the system.^31^ Here, a Mulliken population greater than 0.4 |*e*| is considered a covalent bond.

As geometry optimizations only find local minima on the potential energy surface, these calculations were supplemented with *ab initio* molecular dynamics (AIMD) simulations, to sample the potential energy surface and identify lower energy states. AIMD simulations were carried out using the DFT plane-wave code CASTEP,^27^ employing on-the-fly pseudopotentials and a plane-wave basis set with a cutoff energy of 390 eV. A time step of 0.5 fs was employed, determined through preliminary calculations under the NVE ensemble that ensured a Hamiltonian energy drift of less than 10 meV/atom/ps. The production runs used the NVT ensemble, at a temperature of 309 K (body temperature), maintained using the Nóse–Hoover thermostat.^32^ Simulations were run for 14 ps. Full geometry optimizations were performed at time intervals of 0, 2, 4, 6, 8, 10, 12, and 14 ps during the AIMD simulation to identify local minima on the potential energy surface sampled by AIMD. As with the surface geometry optimizations, the exchange-correlation was described using the PBE-GGA approximation.^28^ The TS dispersion correction^29^ was employed to maintain consistency between calculations and allow comparison between geometry optimizations and the AIMD results. Simulations were carried out by minimizing the total energy per atom to within 10^–3^ eV, using a conjugate gradient algorithm.

## Results and Discussion

### Calcium Oxalate Adsorption on the COD (110) Surface

**Figure 1:**
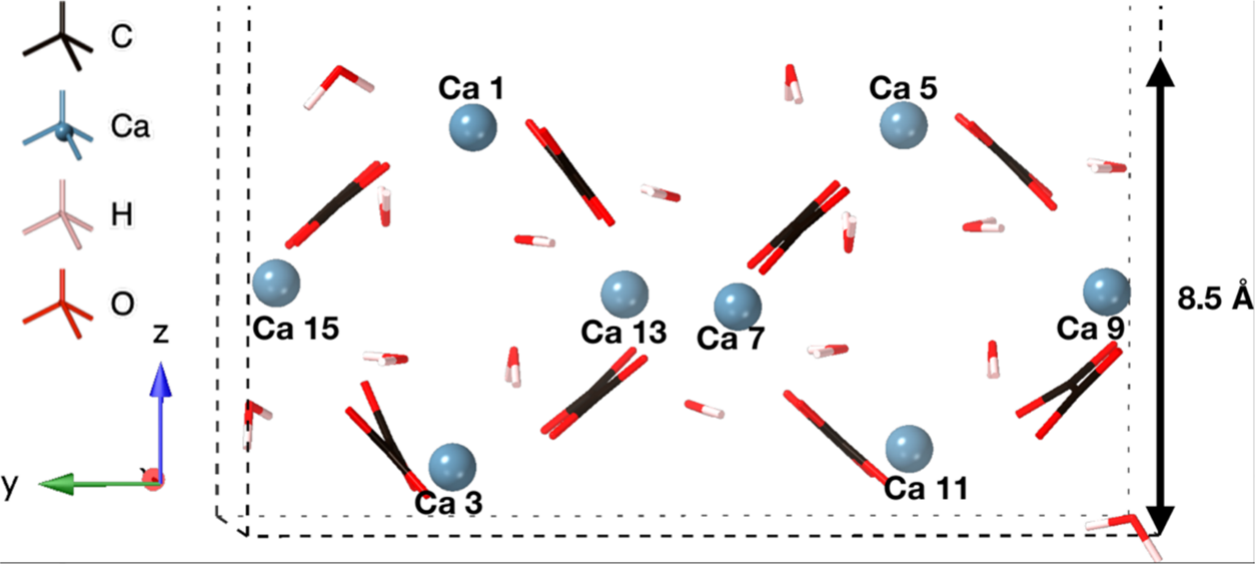
COD (110) surface showing location of calcium 5, 9, and 11 ions. The vacuum is truncated in the *z*-direction for clarity.

In this work, we discuss adsorption onto the COD (110) surface. We specifically refer to calcium ions 5, 9, and 11 (Figure 1). These calcium ions are located at the upper surface of the model (Ca 5), 4 Å from the upper surface, into the bulk (Ca 9), and 8 Å from the upper surface on the lower surface (Ca 11). The calculated surface energy of this surface model was 0.032 eV/Å^2^, in line with the previous study of Debroise et al.^15^ who reported a surface energy of 0.036 eV/Å^2^.

**Figure 2:**
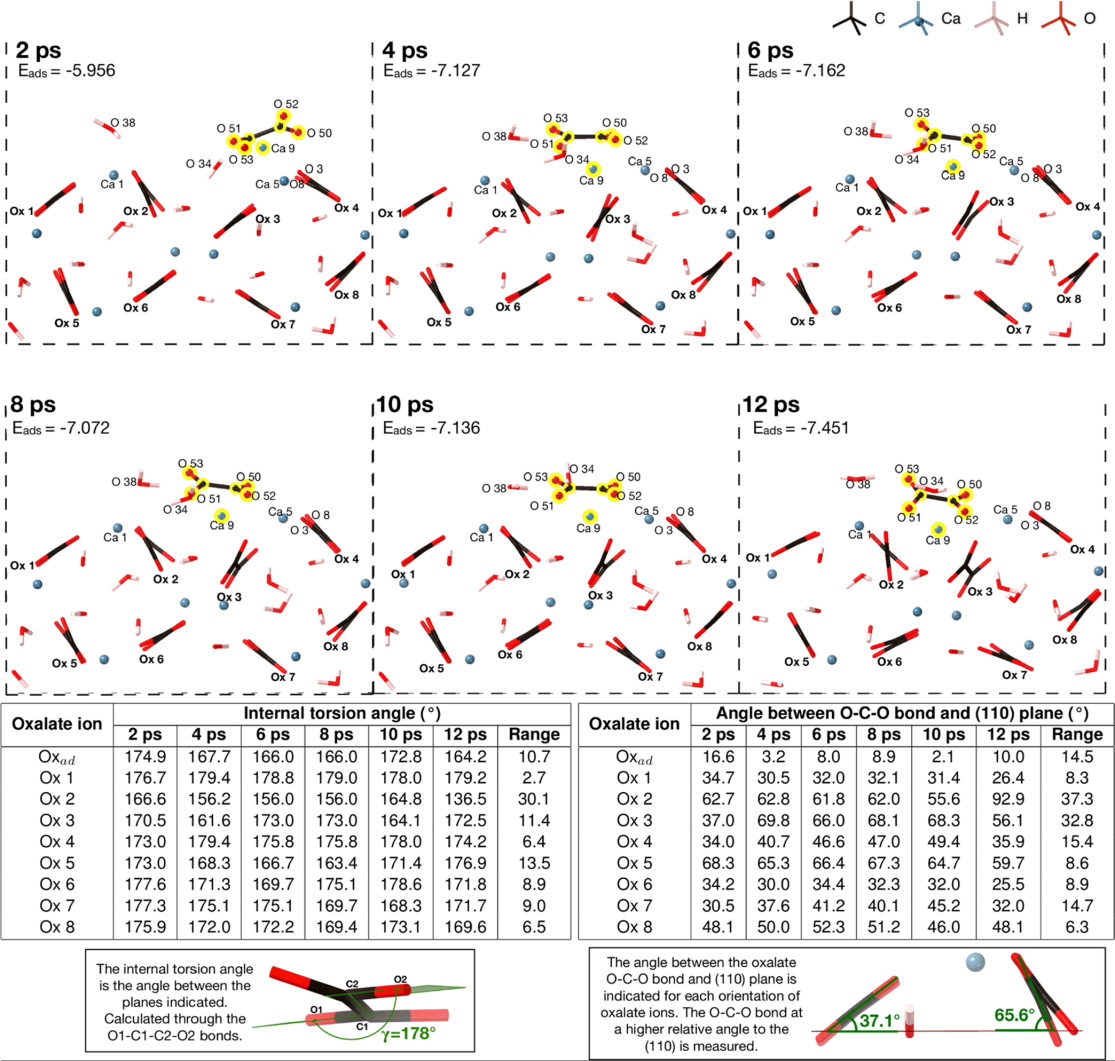
*Ab initio* molecular dynamics adsorption of CaOx_ad_ adsorbing onto the COD (110) surface. The labeled atoms are those involved in bonding and CaOx_ad_ is highlighted in yellow. The internal torsion angles and the angle between the oxalate O–C–O bond and the (110) plane are shown in the tables.

AIMD simulation of calcium oxalate adsorbing (CaOx_ad_) onto the COD (110) surface helps us to understand the process of further crystal growth. At the start of the simulation, the calcium oxalate is positioned 4 Å above the surface (Figure 2) directly above an oxalate ion. Within 2 ps, calcium oxalate interacts with the surface, forming bonds from the oxalate oxygen atoms to surface calcium ions. The system stabilizes after 4 ps of simulation, and chemistry can be inferred from this point forward. The adsorption energies decrease as the simulation progresses, with the lowest energy (−7.446 eV, −0.065 eV/atom) binding conformation occurring at 11.990 ps, suggesting the most stable position has been achieved.

Oxalate 2 (Ox 2, Figure 2) experiences the greatest change in both internal torsion angle and the angle between the O–C–O bond and the (110) plane, as the adsorbing species move closer to it throughout the simulation. As can be seen in Figure 2, at 2 ps, Ca 9 is located above Ca 5 and Ox 2 is undisturbed in its crystallographic position, with a similar angle between the O–C–O bond and the (110) plane as that of Ox 5. Between 4 and 10 ps, Ox 2 is attempting to maintain its internal torsion angle; however, at 12 ps, when Ca 9 coordinates and a lower energy configuration is found, the Ox 2 internal torsion angle has changed by 30° since its configuration at 2 ps.

**Figure 3:**
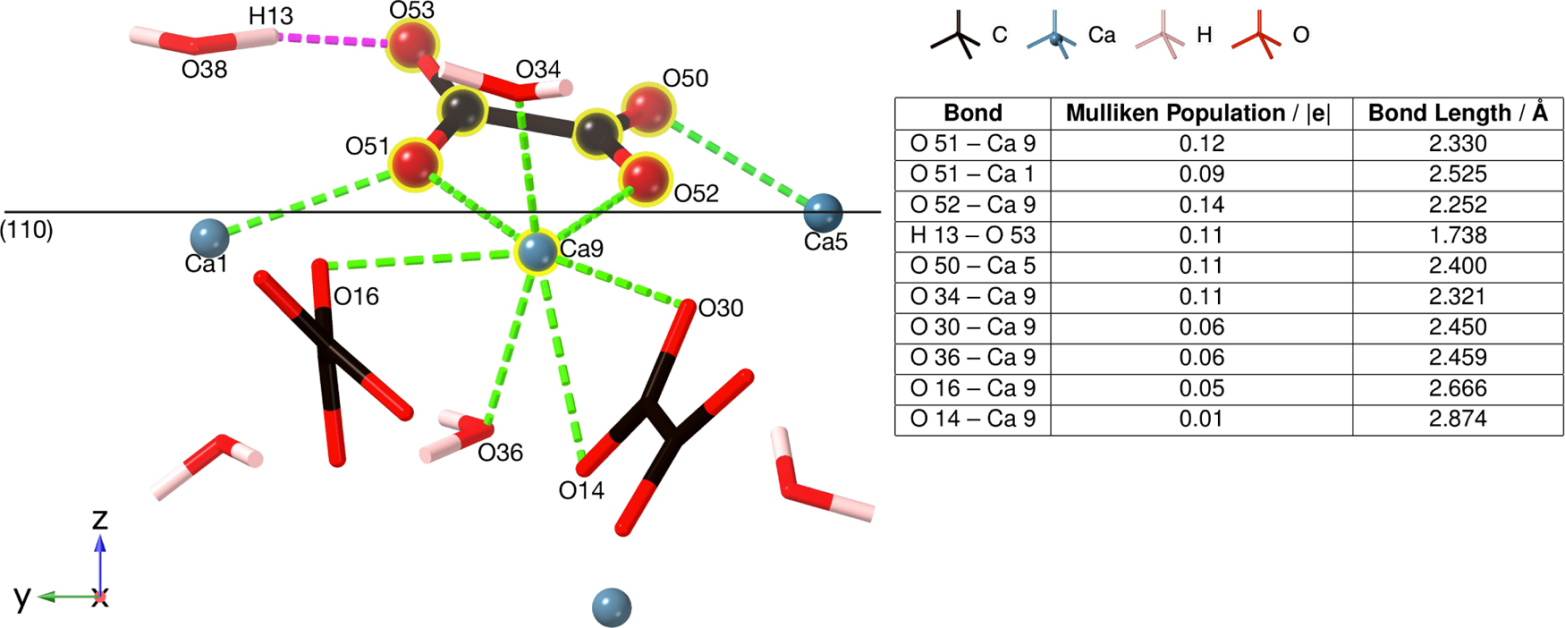
Geometry optimization of calcium oxalate bound to the COD 110 surface at 11.990 ps time step. Ionic bonds are shown as dashed green lines and the hydrogen bond with a dashed pink line. The table shows the new bonds and their populations.

The angle between the O–C–O bond and (110) plane (Figure 2) for Ox 7, on the lower surface, deviates by 14.7° over the course of the trajectory, which allows Ox 3 (above it) to move further into the bulk and Ca 9 to penetrate the surface. This illustrates the impact of calcium oxalate adsorption on the upper surface, extending throughout the model. The adsorbed oxalate ion binds to the surface via four ionic bonds (0.11 |*e*|, 0.14 |*e*|, 0.12 |*e*|, and 0.09 |*e*|) to surface calcium ions. Interestingly, the adsorbed oxalate binds to the surface calcium ions more strongly, with an average Mulliken population of 0.12 |*e*|, than the oxalate ions within the model structure bind to calcium ions in the clean surface (average bond population: 0.10 |*e*|), indicating favorable extension of the COD (110) surface. Ca 9 penetrates the surface structure, settling into a position 0.006 Å lower in the surface than Ca 1, and 0.017 Å lower than Ca 5. The adsorbed oxalate ion lies at an angle of 10° to the (110) surface (Figure 3), which is an unusual position (parallel to the plane) compared to the native oxalate ions. From geometry optimization alone, the adsorbed oxalate lies at an arguably more realistic angle of 66° to the (110) plane. It would be interesting to observe whether this oxalate ion would eventually adopt a regular crystallographic position in longer time scale classical MD simulations.

By 12 ps, atom movements within the bulk of the surface can be seen (Figure 3), with the top layer of the surface having moved significantly. Oxalate ions at the top surface increase their internal torsion angles, while those at the lower surface experience less deviation.

Overall, the adsorbing Ca 9 binds to five oxalate oxygen atoms and two oxygen water atoms, continuing the crystallographic structure of the crystal and growing the surface. The free oxalate binds to the surface via five ionic bonds, four of which are to calcium ions (Ca 5, Ca 1, and Ca 9) and one to a water molecule (H 13).

### Phosphocholine Headgroup

**Figure 4:**
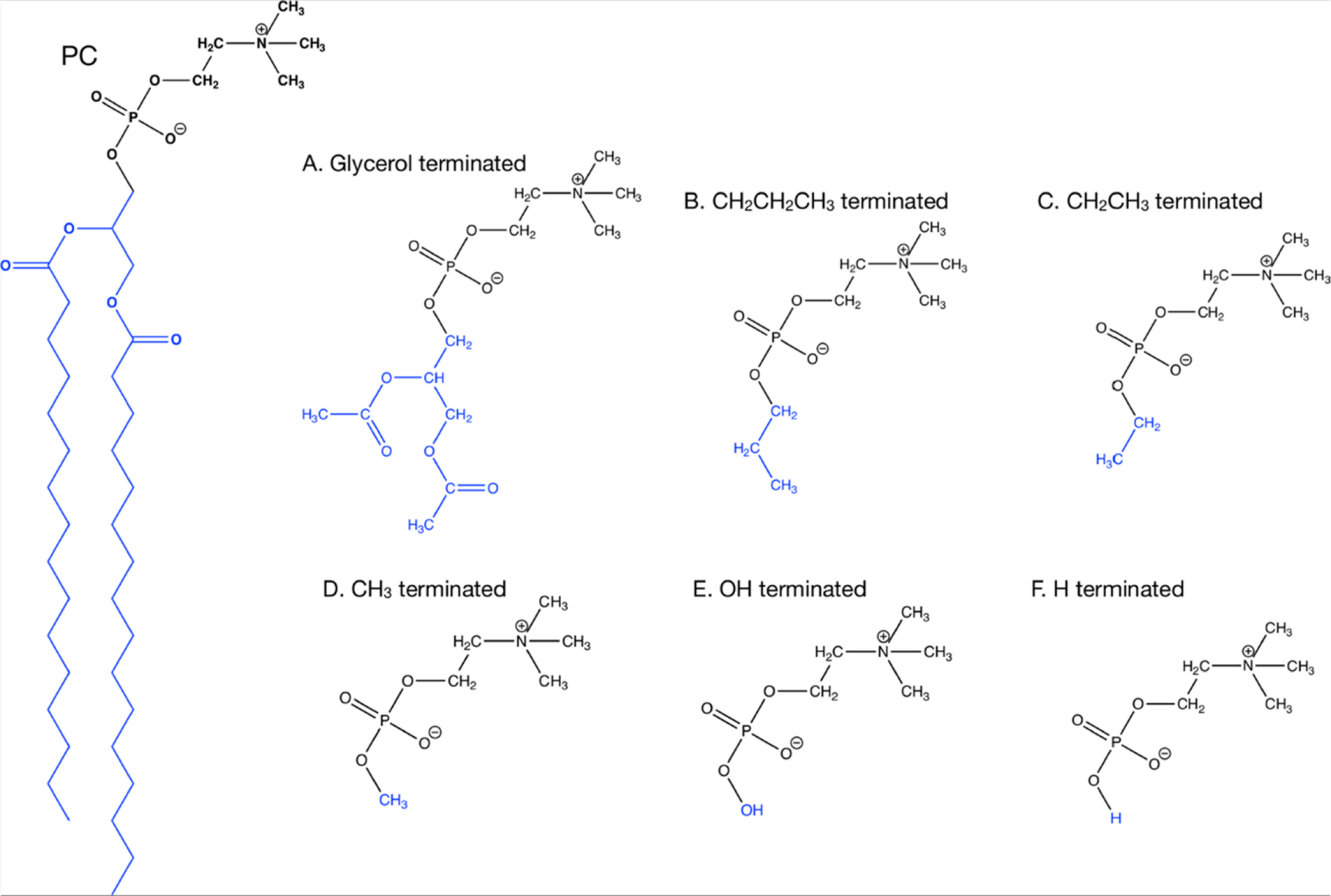
Chemical structures of the phosphocholine model terminations.

**Figure 5:**
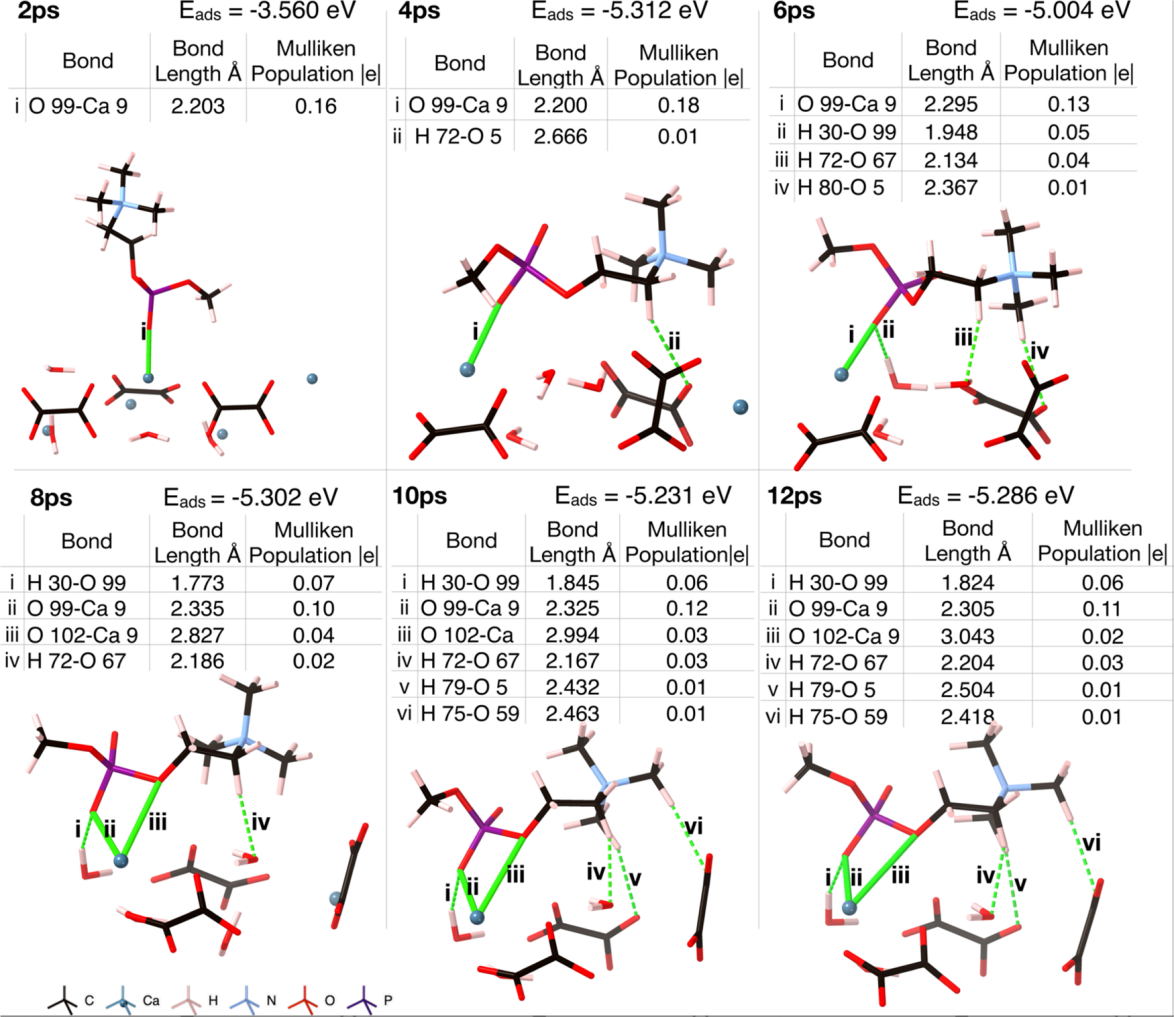
AIMD of the PC headgroup binding to the COD (110) surface. Ionic bonds are shown as solid green lines, and hydrogen bonds as dashed green lines.

To model the chemical interactions effectively and efficiently, the phosphocholine structure was truncated, while retaining all the chemistry necessary to understand its interactions with the surface structure. Therefore, the PC headgroup was cleaved between the phosphate and the glycerol. The impact of different terminating groups on the charges and bond lengths of the final phosphate ion of the PC headgroup were studied (Figure 5). The first model termination (A in Figure 4) is representative of the glycerol-attached tail, and each tail is terminated with a methyl group after the ester linkage. The remaining model terminations truncate the structure at the headgroup phosphate. B to D (Figure 4) investigate the length of a carbonyl chain; B simplifies the termination to a propyl group, C an ethyl group, and D a methyl group. Model E simplifies the termination further to a hydroxyl group and F to a hydrogen. Each configuration was geometry-optimized, and the charges on the phosphate and its adjacent oxygen atoms were investigated along with the bond lengths (Supporting Information, Figure S2).

There was little impact on atomic charges using alkyl chains as termination groups (Supporting Information, Figure S2B–D). The charges on each atom remained within 0.4 |*e*| of the glycerol termination. Termination D gives the best representation of the atomic charges in model A, as each atom deviates by only 0.02 |*e*|. By contrast, for the alcohol-terminated phosphocholine headgroup (*E*), the oxygen atom’s atomic charge (O 3) adjacent to the termination became more negative, −1.06 |*e*|, in comparison to −0.77 |*e*| for A. This is due to the alcohol group being a stronger electron-donating group than an alkyl chain. O 3 also became more negatively charged (−1.05 |*e*|) in model F (hydrogen termination). In model F, the O 3 becomes part of an alcohol group rather than part of a phospho-ester bond.

Next, the bond lengths of the phosphorus–oxygen bonds were compared, along with the bond length from C 1 to 4 (Supporting Information, Figure S2). In model E (hydroxyl group), the C 1–O 4 bond length varies by 0.064 Å in comparison to model A. For model F (hydrogen), the difference is only 0.003 Å, which is similar to model D (methyl), which varies by just 0.002 Å. From analyzing the bond lengths, both models D and E provide similar results to model A, suggesting either structure would be a sufficient simplification of the terminating group. Lastly, the Mulliken populations give an indication of bond strength. This is important to consider as a stronger electron-donating group may decrease the bond strength further along the molecule. Like the atomic charges and bond lengths, there is a negligible difference between models B to D. Each model has C–O Mulliken populations within 0.02 |*e*| of model A. Model E has a lower Mulliken population (0.47 |*e*|) for the C 1–O 4 bond than in model A (0.53 |*e*|), again demonstrating that it is less effective at representing the atomic interactions. Model F also models the Mulliken populations closely, remaining within 0.04 |*e*| of model A. The best termination would therefore be model D, with the methyl group. This termination effectively models the chemical interactions in the same way as model A, but has 18 fewer atoms, which is important for these high-cost AIMD calculations.

### AIMD Simulations of Phosphocholine Headgroup

**Figure 6:**
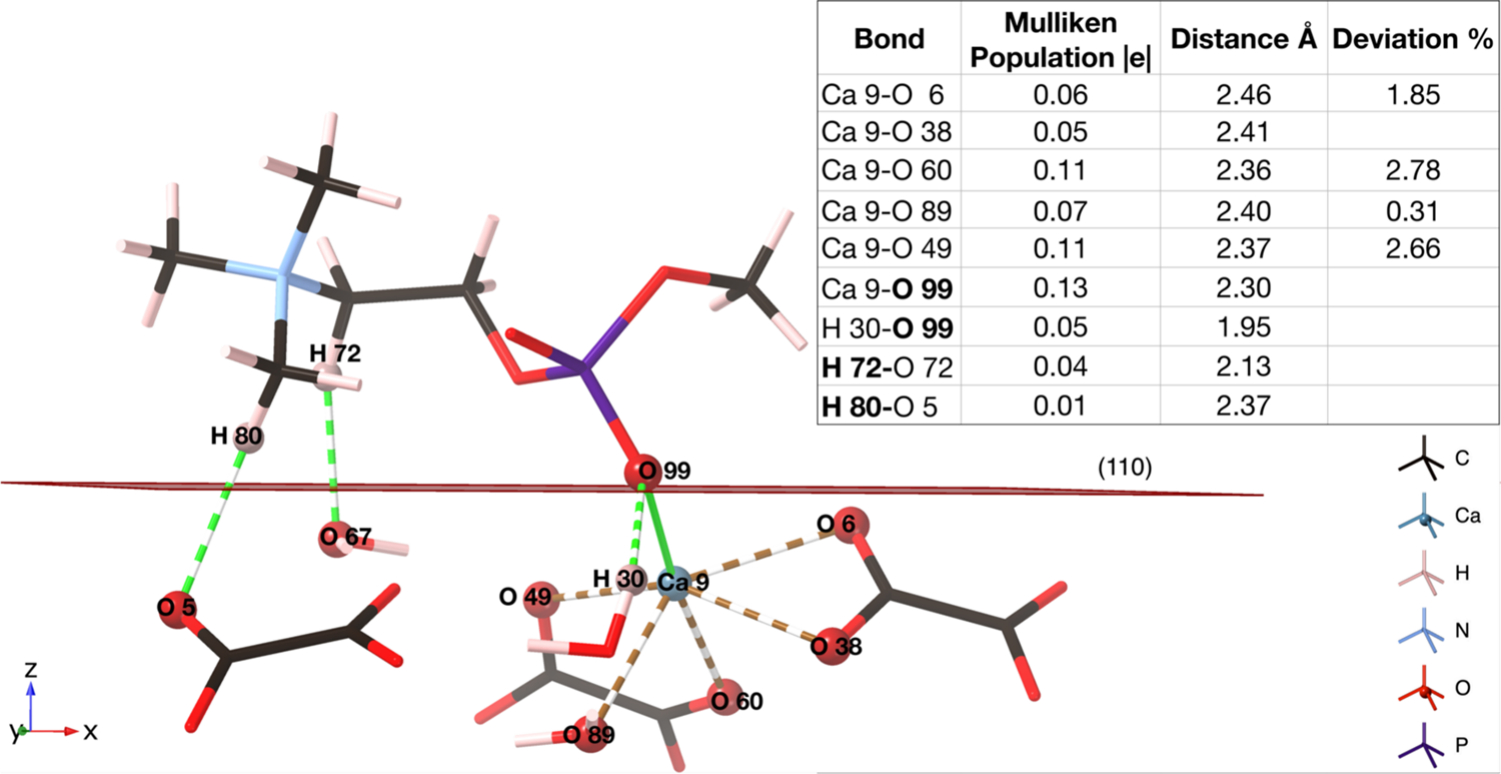
Bonding of the phosphocholine headgroup to the surface at the 6 ps time step, *E*_ads_ = −5.004 eV. Green lines represent ionic bonds from phosphocholine to the surface, and green dashed lines represent hydrogen bonds from phosphocholine to the surface. Brown dashed lines represent bonds from Ca 9 to oxalate and water. The table shows the bonding analysis.

The phosphocholine headgroup adsorbs favorably onto the COD (110) surface after 2 ps of AIMD simulation (Figure 5). As the simulation progresses and lower energy states are found, the phosphocholine rearranges on the surface. At first, the phosphate group binds to the surface with the amine group perpendicular to it. After 2.7 ps, the amine collapses onto the COD (110) surface, where it forms a hydrogen bond to surface water; here, the adsorption energy of the phosphocholine to the surface is −3.943 eV (−0.0165 eV/atom). After 6 ps, the phosphocholine headgroup has formed more hydrogen bonds to the surface (Figure 6), and the adsorption energy is −5.004 eV (−0.0207 eV/atom). The adsorption is dictated by an ionic bond between Ca 9 on the surface and O 99 on the PC, with a Mulliken population of 0.13 |*e*|. This bond population is equal to that of the Ca–O (water) bonds on the adsorbate-free surface but has a shorter bond length (2.30 Å compared to 2.40 Å on the adsorbate-free surface). The new bond to Ca 9 is very similar in strength to other bonds made by this ion. For example, Ca 9 binds to O 89 of a surface water molecule, with a population of 0.10 |*e*| on the adsorbate-free surface and 0.07 |*e*| on the surface with phosphocholine adsorbed. Furthermore, Ca 9 binds to the oxygens (O 49, O6) in oxalates with populations of 0.10 and 0.13 |*e*| on the adsorbate-free surface and 0.11 and 0.06 |*e*| on the surface with phosphocholine adsorbed. This result shows that phosphocholine is bound to the surface with a similar degree of stability as the bulk water molecules and oxalate ions within the crystal structure.

**Figure 7:**
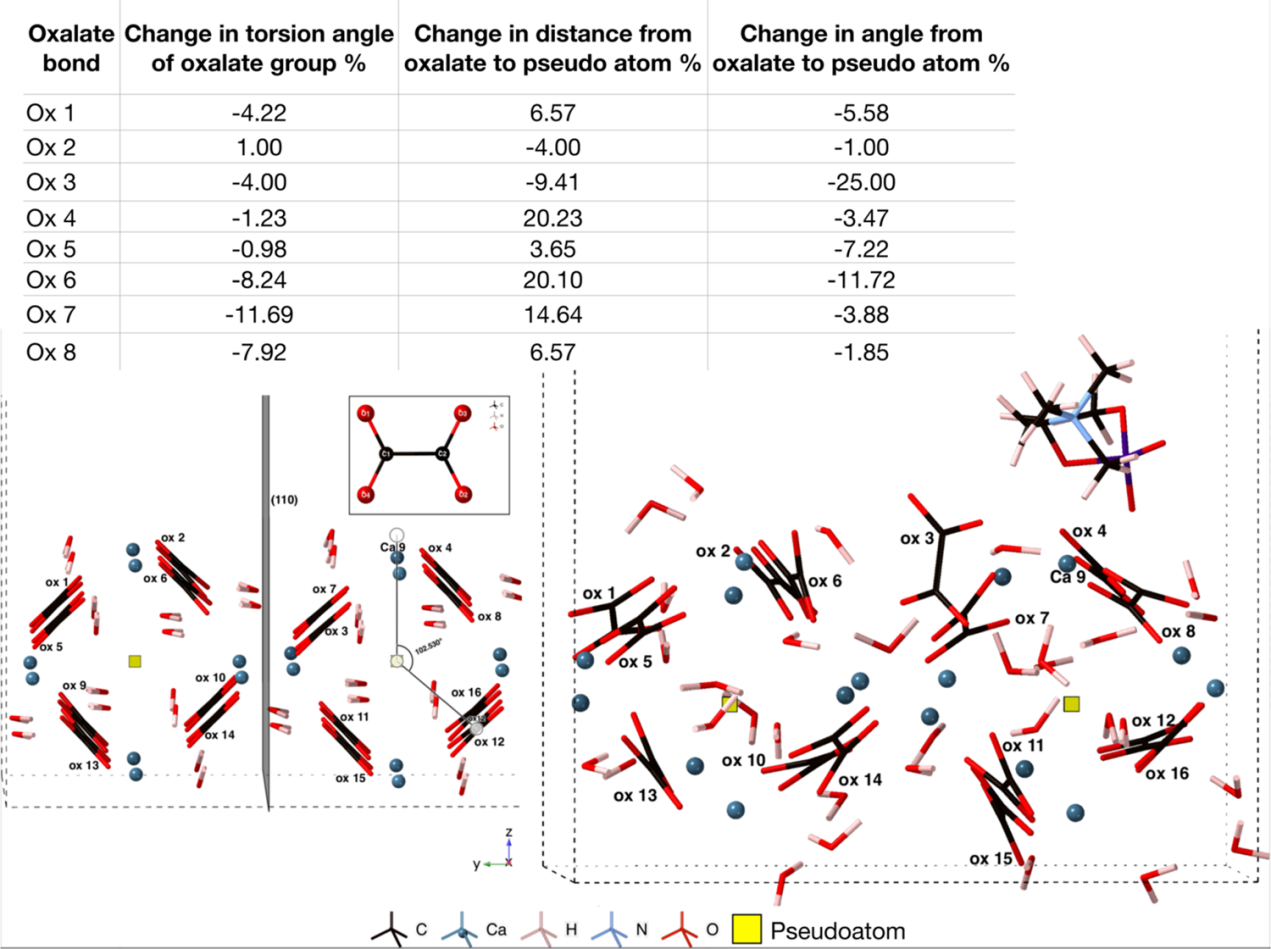
(Left) Bonding within the adsorbate-free COD (110) surface. (Right) Bonding of the phosphocholine headgroup to the COD (110) surface. Yellow squares represent the pseudoatom used as a reference for oxalate group movement. The gray plane represents the (110) surface. The inset shows the oxalate group with numbered atoms; torsion angles were measured from O1–C1–C2–O2 for each group. The angle shown is an example of the angle from oxalate to the pseudoatom. The table shows movement of oxalate groups on the surface.

The bound phosphocholine headgroup is causing the atoms to contract in the <110> direction, demonstrated by the contraction of Ca–O bond lengths. Ca 9–O 49, Ca 9–O 60, and Ca 9–O 6 bond lengths all decrease by over 1.5% (Figure 6). This puts significant tension on the crystal structure and causes the bulk structure to lose symmetry. This can be clearly seen in Figure 7, which shows the adsorbate-free COD (110) surface and the surface once PC has been adsorbed, for comparison. The associated table shows the change in position of the oxalate groups after the phosphocholine headgroup adsorbs onto the surface. A reference point pseudoatom was placed centrally in each cavity of the unit cell (Figure 7) to determine the relative movements of all atoms within the model, and the change in the torsion angle describes the movement within each oxalate and the loss of internal symmetry. The phosphocholine adsorbs onto Ca 9, and as expected the nearest oxalate groups, 3, 4, 7, and 8, demonstrate the greatest movements, along with oxalate 6. The torsion angle of oxalate 7 changed by over 10%, suggesting a large movement of the oxygen atoms in relation to the carbon atoms, and oxalates 4, 6, and 7 increased in distance from the reference point by over 15%. This is a direct consequence of the Ca 9–O 60 bond increasing in length by 2.6%, moving away from the reference point, and allowing O 99 better access to Ca 9. This change is indicative of the disruption to the long-range intermolecular forces, after the phosphocholine adsorbs. The phosphocholine also causes oxalates 3 and 4 to be pushed away from Ca 9, and subsequently their reference point, and pushed up toward the surface, which consequently causes oxalate 6 to be pushed away from its reference point in the <110> direction. Oxalates 3 and 6 decrease their angle to the reference point by over 10% after the phosphocholine headgroup adsorbs. The decrease in angle indicates that the oxalate groups have become more parallel to the <110> plane.

The adsorption energy of phosphocholine to the surface is favorable, −5.004 eV (−0.0207 eV/atom), suggesting that even though the symmetry of the crystal is lost, the interaction with phosphocholine is still favored. In comparison to the adsorption energy of further calcium oxalate growth (Figure 4, −7.446 eV, −0.0653 eV/atom) the phosphocholine headgroup binds to the COD (110) surface less favorably, suggesting it is less likely to bind when in direct competition with calcium oxalate, but if it is within proximity of a COD (110) surface, it will bind favorably.

The significant disruption of the COD (110) surface crystal structure following PC adsorption during the simulation suggests that if PC were to adsorb onto COD crystals, the crystal habit may undergo a significant change. Crystal habit modifications have previously been observed with COD crystals growing in the presence of adsorbates such as poly(acrylic acid), where crystals grew in a dumbbell-like habit^11^ and glutamic acid, where the (100) surface became elongated.^12^ Furthermore, Chien et al.^10^ adsorbed polyaspartic acid, neutrally charged peptides, and OPN-ASARM peptides, onto COD crystals. The OPN-ASARM peptides are synthesized peptides of osteopontin,^10^ which are acidic, with serine- and aspartate-rich motifs. The peptides contain clusters of phosphate and carboxylate groups with a high density of negative charge. Chien et al.^10^ found that the crystal habit of the COD crystals changed with each adsorbate. The nonphosphorylated residues of aspartate-rich peptides altered the habit of the COD crystals to pseudododecahedral, which contrasts with the phase-pure dipyramidal crystal habit. The phosphorylated OPN-ASARM residues with 3 and 5 phosphoserine residues formed crystal spherulites that displayed mushroom- and dumbbell-shaped habits. The spherulites grew as layered platelet structures, with additional crystals growing and repeating their structures. Based on the underlying COD structure, it was suggested that the phosphate groups of the organic residues were binding to surface calcium atoms and thus playing a key role in the crystal habit changes. This was also observed in our work with the PC phosphate directing the adsorption via its electrostatic interaction with the surface. Furthermore, an adsorption energy (−2.696 eV) was previously calculated for the ASARM 5 peptide (DDpSHQpSDEpSHHpSDEpSDEL), using molecular dynamics, which showed favorable binding to the COD (110) surface.^10^ This result agrees with the results presented here, where we found the binding of the PC headgroup to be favorable (−5.312 eV).

These calculations indicate that crystals can nucleate on the outer membranes of the kidney epithelial cell walls on the exposed PC headgroups. They also show that cleaved phosphocholine groups, which are at elevated levels in the urine of stone sufferers,^18^ can bind to growing calcium oxalate crystals.

### Phosphocholine-Induced Calcium Oxalate Growth

The phosphocholine headgroup binds favorably to the COD (110) surface, showing that this macromolecule could initiate COD crystal agglomeration on the epithelial wall of the kidney tubule. However, could the binding of phosphocholine promote further calcium oxalate growth of crystals formed within the urine? To investigate this, the bound state above (Figure 6) was used to run a dynamics simulation with a calcium oxalate molecule adsorbing onto the modified surface.

**Figure 8:**
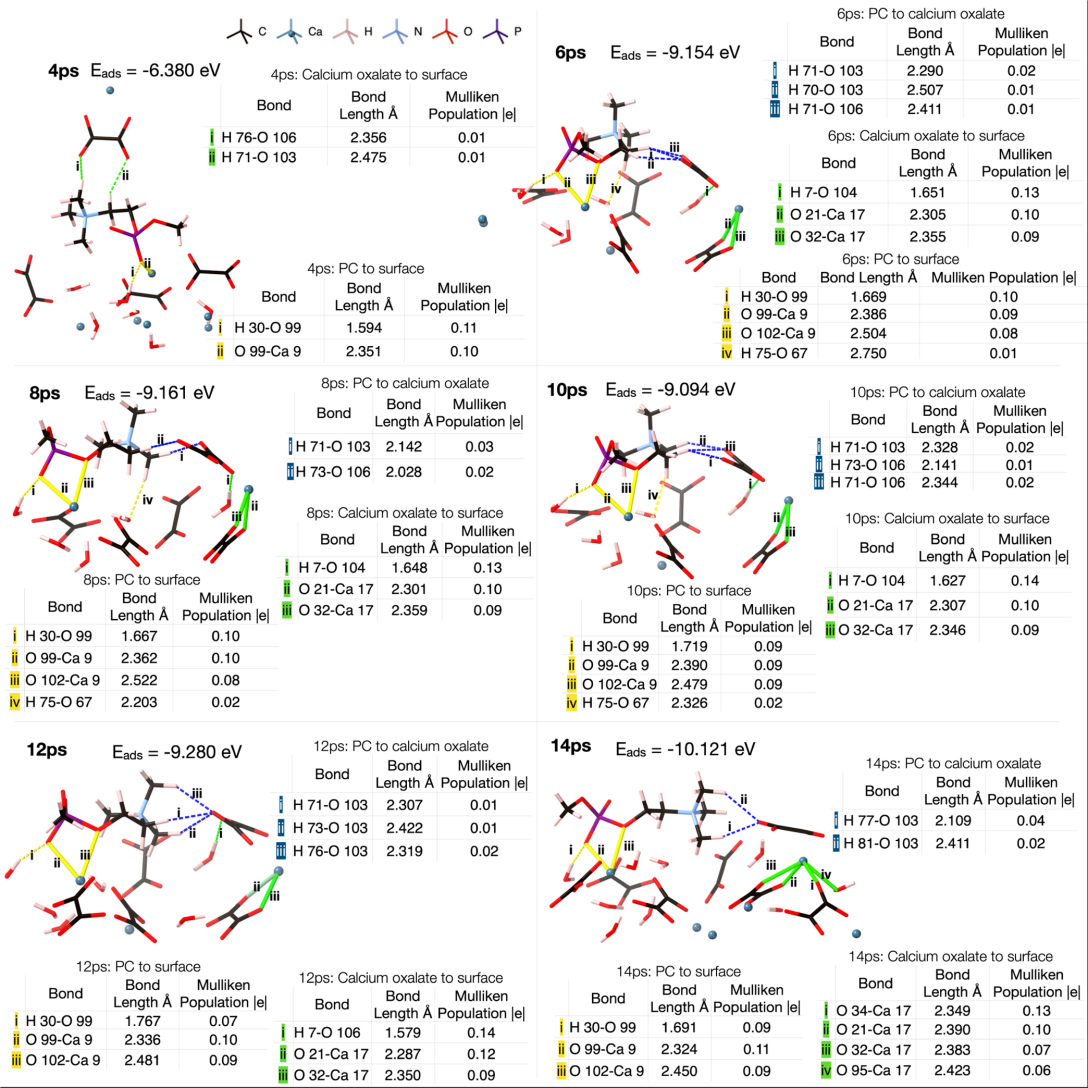
AIMD of CaOx adsorbed on the PC-bound COD (110) surface over 12 ps of simulation. Yellow bonds represent bonds from PC to surface, blue bonds from PC to CaOx, and green bonds are between CaOx and the surface.

After 4 ps of AIMD simulation (Figure 8), the calcium oxalate is bound to the phosphocholine with an adsorption energy of −6.379 eV (−0.0256 eV/atom) and after 12 ps, the adsorption energy decreases further to −9.280 eV (−0.0373 eV/atom). The lowest energy binding configuration occurred at 10.9365 ps, with an adsorption energy of −9.423 eV (−0.0378 eV/atom).

**Figure 9:**
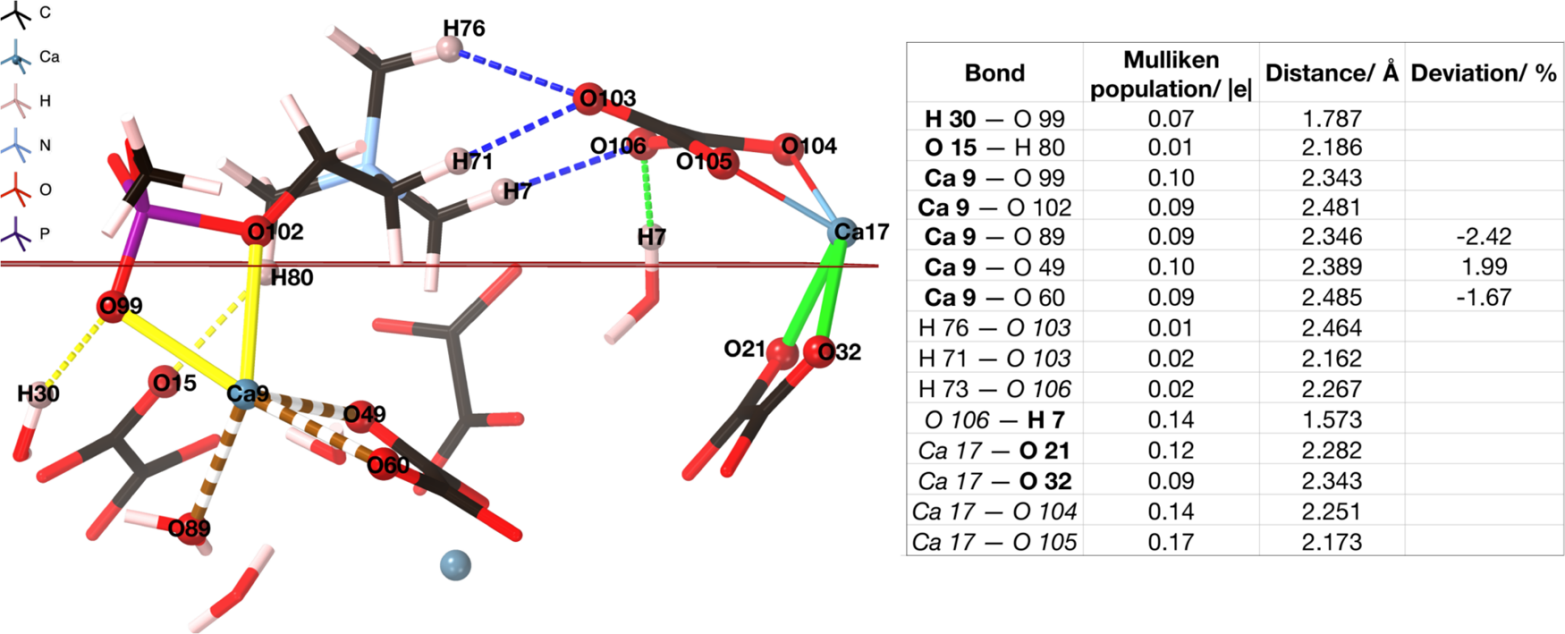
Bonding of CaOx adsorbing onto the phosphocholine (PC)-bound COD (110) surface at 10.94 ps. Blue dashed lines represent hydrogen bonding from additional CaOx to PC. Green lines represent ionic bonds from CaOx to the surface, and green dashed lines represent hydrogen bonds. Yellow lines represent ionic bonds from PC to the surface, and yellow dashed lines represent hydrogen bonds. Brown dashed bonds represent bonds from Ca 9 to the surface. The table shows bonding analysis, where atoms within the surface are shown in bold typeface.

In the lowest energy configuration (Figure 9), the oxalate group binds to the phosphocholine via three hydrogen bonds, H 76, H 71, and H 73 (0.01, 0.02, 0.02 |*e*|), and to the surface via one hydrogen bond from 106 to a water molecule (H 7). This is a relatively short hydrogen bond, with a length of 1.573 Å and a population of 0.14 |*e*|. The newly introduced calcium ion binds to the surface via two ionic bonds to a surface oxalate, specifically O 21 (2.282 Å, 0.12 |*e*|) and O 32 (2.343 Å, 0.09 |*e*|).

Figure 9 shows that the phosphocholine group remains tightly bound and is not readily displaced by incoming calcium oxalate. Nevertheless, the bond strength between Ca 9 and O 99 has decreased slightly, following calcium oxalate adsorption, from 0.13 |*e*| (with only the PC-bound) to 0.10 |*e*|. However, an additional ionic bond was formed from Ca9 to O 102 of the PC, with a population of 0.09 |*e*|. This contributes to an overall increase in the bond strength of PC to the surface. The low adsorption energy shows that it is favorable for calcium oxalate to grow on the PC-bound COD (110) surface, and further illustrates the extremely stable binding of the PC headgroup. Notably, it is more favorable for the calcium oxalate to bind to the phosphocholine-bound COD surface (−9.423 eV, −0.0378 eV/atom), than it is to the adsorbate-free (110) surface (−7.446 eV, −0.0653 eV/atom), highlighting the capability of the bound phosphocholine to encourage further calcium oxalate crystal growth. This result validates those of Valido et al.^33^ who observed, using FTIR on kidney stone samples, the presence of lipids that appeared to stabilize the COD crystallites.

### COD (110) Surface Sandwich with Phosphocholine Headgroup

Microscopy of kidney stones has revealed detailed information about their structure, with concentric rings of COM and COD growing around a central nidus.^14^ For these concentric rings to grow, the COD crystals must be free of charge in the urine. Furthermore, super-resolution autofluorescence imaging (SRAF), shows organic matter-rich and mineral-rich layers forming, as well as empty pores thought to have contained organic matter that has degraded.^14^ This is particularly pronounced when employing transmitted light polarization and phase contrast (CPOLPC), where the organic material shows up as a darker layer in between brighter mineral deposits.^14^ This leads to the question of whether phosphocholine, as a component of that organic layer, could enhance further mineral growth. Expanding on the previous calculations, which suggest crystal growth above the bound phosphocholine is very favorable, we created a model of two crystallites approaching one another. In this system, the phosphocholine headgroup was placed in between two COD (110) surfaces, and AIMD simulations were carried out.

**Figure 10:**
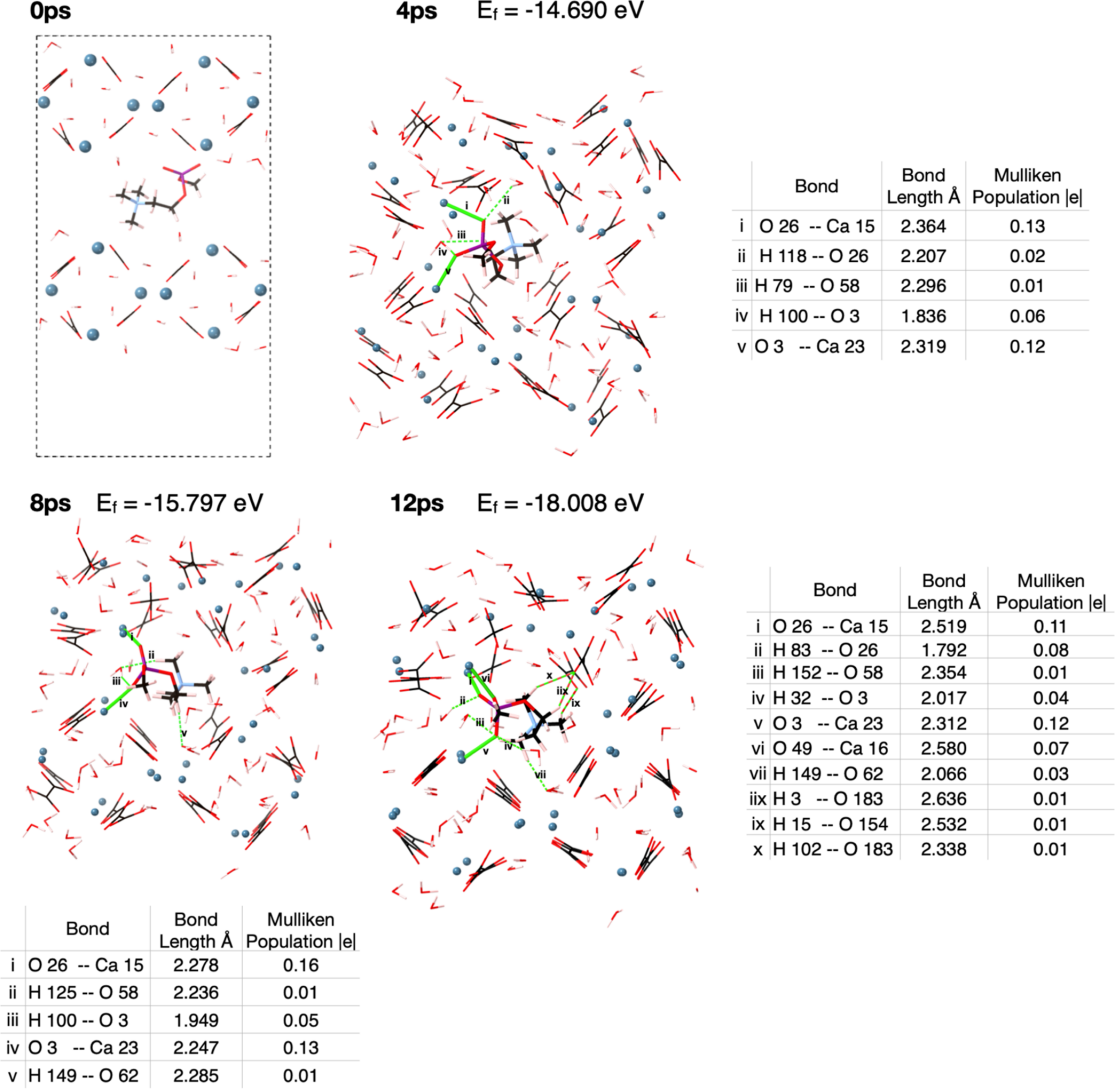
AIMD of PC between two COD (110) surfaces from 0 to 12 ps. Green lines represent ionic bonds, and green dashed lines represent hydrogen bonds. The formation energies, E_f_, calculated using eq 1, are given in each pane, and the inset tables show bonding analysis at 4, 8, and 12 ps.

**Figure 11:**
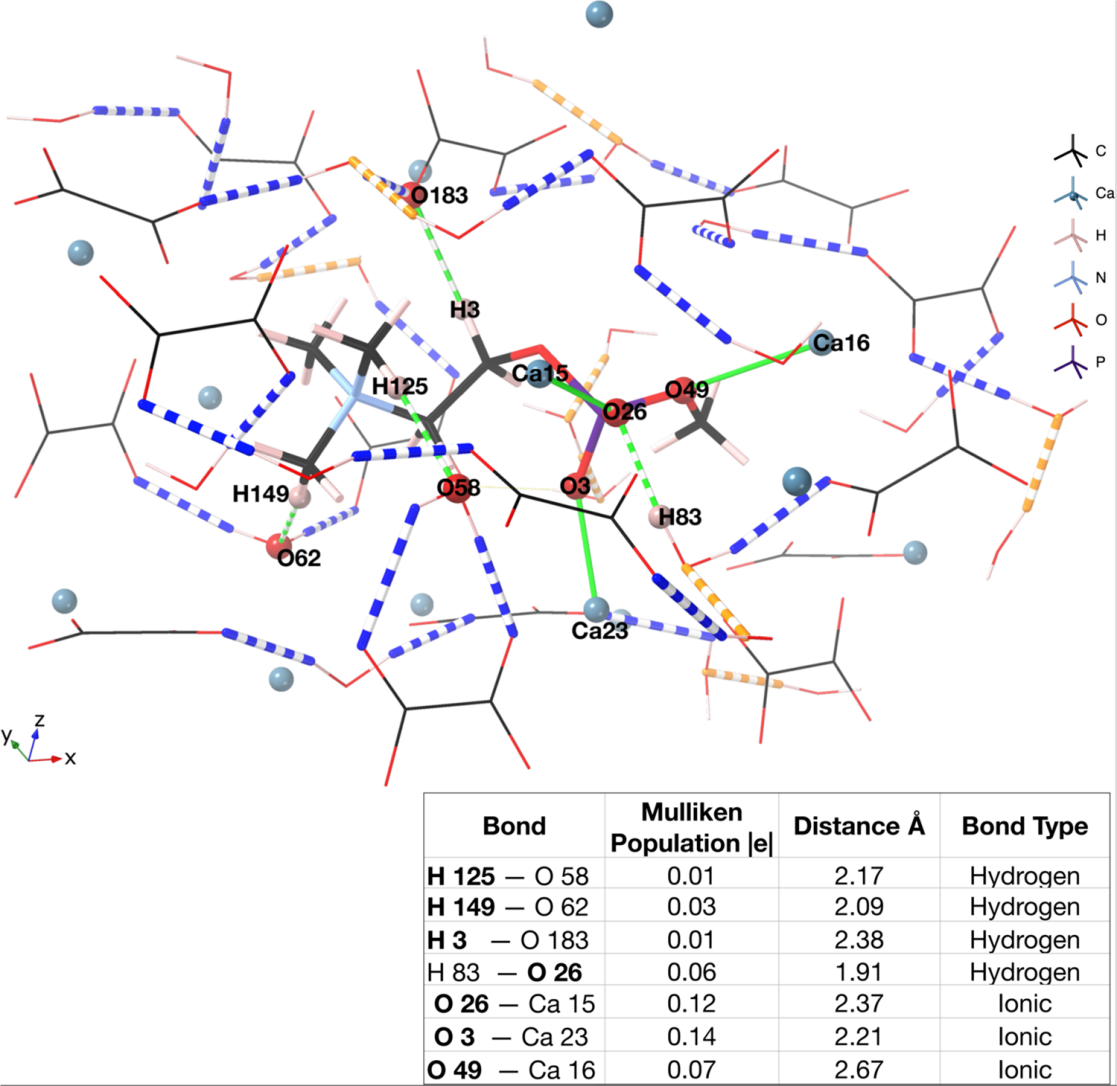
COD (110) surface with phosphocholine sandwiched between layers. Ionic bonds between phosphocholine and the mineral are shown as green bonds, and hydrogen bonds are represented by dashed green bonds. Hydrogen bonds between water molecules and oxalate groups in the mineral are shown as dashed blue bonds. Hydrogen bonds between water molecules are shown by dashed orange bonds. The table shows bonding analysis, where atoms in the phosphocholine are shown in bold typeface.

At first, the surfaces pull apart, away from the enclosed PC, but after 1.7 ps, the surfaces begin to encapsulate the PC headgroup (Figure 10). After 10 ps of simulation, the two COD (110) surfaces surround the PC headgroup and bonds are formed from both the upper and lower surfaces (Figure 11). The ionic bonds formed between calcium and oxygen (0.14, 0.12, and 0.07 |*e*|, Figure 11) have a similar strength, determined by Mulliken population analysis, as the Ca–O bonds within the crystal structure of the adsorbate-free COD (110) surface (0.10 and 0.13 |*e*|). This indicates that the phosphocholine headgroup is being held stably within the crystal structure.

The water network remains unchanged with each oxalate group coordinated to two water molecules (blue bonds in Figure 11) and the water molecules hydrogen-bonded to one another (orange bonds in Figure 11). The two strongest ionic bonds (0.14 and 0.12 |*e*|) form between the oxygen atoms of the phosphocholine phosphate and nearby calcium ions. These populations compare to the average Ca oxalate population of 0.11 |*e*| in the COD (110) surface. This suggests that the inclusion of phosphocholine does not substantially weaken the bonding within the crystal structure. The feasibility of this encapsulation is validated by the formation energy (calculated in the same manner as the adsorption energy, eq 1); it is highly favorable for the phosphocholine to be captured between the two surfaces, with a formation energy of −18.008 eV (−0.0396 eV/atom). In summary, this indicates that phosphocholine binding two crystals of calcium oxalate dihydrate together through their respective (110) surfaces is a likely and favorable scenario and one that may be likely to occur within the urine.

### Control Calculation

To confirm the promoting effect of PC on the aggregation of COD (110) surfaces, an AIMD simulation was carried out with two COD (110) surfaces 6.7 Å apart. The energy of the two COD (110) surfaces aggregating was −10.478 eV at 7 ps (Supporting Information, Figure S3), whereas the energy of the aggregation with PC present was −18.008 eV (Figure 10). This result confirms that PC does indeed promote the aggregation of the COD (110) surfaces by −7.53 eV, in addition to disrupting the crystallographic symmetry.

Experimental work on phospholipid monolayers (including phosphocholine layers)^34^ has shown that increased mobility in the monolayer results in more effective crystallization, as the molecular rearrangement permits better, lower energy, interactions with calcium ions. From a biological perspective, this makes nucleation of calcium oxalate on cellular membrane material in the urine highly likely and favorable. It has been shown that the rate of excretion per hour of leukocytes and nonsquamous epithelial cells to the urine is in the region of 18,000–196,000 per hour, thus providing abundant material within the urine for nucleation to take place.^35^

## Conclusions

To date, there has been little research into understanding the inorganic–organic interactions that occur in kidney stones. While it has been shown that calcium oxalate dihydrate (COD), a major component of kidney stones (particularly in stones formed as a result of calcinuria), interacts directly with organic matrix material, there have been few investigations into its role in stone formation. In this work, we have harnessed published data acquired from kidney stone patients, which showed that phospholipids are at higher concentrations in stone sufferers’ urine than in healthy patients’ urine,^18^ to explore the interactions of phosphocholine and the prevalent calcium oxalate dihydrate (110) surface.

Using first-principles molecular dynamics, we found that the PC headgroup is favorably bound to the COD (110) surface (*E*_ads_ = −5.312 eV). The observed growth of the COD (110) surface following this surface interaction revealed that the PC headgroup was actively promoting surface growth, with additional CaOx adsorption being more favorable, (*E*_ads_ = −9.423 eV) than on the adsorbate-free COD (110) surface, (*E*_ads_ = −7.446 eV). We further found that two COD (110) surfaces can successfully and favorably (*E*_ads_ = −18.008 eV) encapsulate a PC headgroup and that the resulting structure is stabilized by the lipid, demonstrating that organic matter can act as a promoter of kidney stone growth and aggregation, having the capacity to “glue” the mineral layers together. Furthermore, this work demonstrated that the encapsulation of PC resulted in a more favorable agglomeration of the COD (110) surfaces, suggesting that PC could be an effective promoter of crystal growth within the luminal space of the kidney tubules. Our results suggest that by inhibiting the ability of phosphocholine to bind to the COD (110) surface, it could be possible to stop the residual effects of increased phosphocholine levels in stone suffers’ urine. As such, the growing COD crystals and the free-floating phospholipids represent potential targets for future drug development.

## Data Availability

All data will be made available from the corresponding author upon reasonable request.

## Supporting Information Available

COD unit cell structure, with the zeolitic water positions highlighted (Figure S1); detailed Mulliken Populations of the truncated phosphocholine structure and all its termination models (Figure S2); and the results of the control simulation of two COD (100) surfaces approaching one another (Figure S3) (PDF)
