# Supplementary material for: Ab Initio Molecular Dynamics Simulations of Phosphocholine Interactions with a Calcium Oxalate Dihydrate (110) Surface
Source: Cryst Growth Des. 2024 Sep 18;24(19):8063–75. doi: 10.1021/acs.cgd.4c01032 (PMC11450748; doi:10.1021/acs.cgd.4c01032)
Supplement: Supplementary file 1 — cg4c01032_si_001.pdf [file cg4c01032_si_001.pdf]

Supporting Information

Ab initio molecular dynamics simulations of phosphocholine interactions with a calcium oxalate dihydrate (110) surface.

Rhiannon Morris,[†] Helen F. Chappell,[†] Andrew J. Scott,[‡] Antonia Borissova,[‡] and James Smith*[†]

†*School of Food Science and Nutrition, University of Leeds*

‡*School of Chemical and Process Engineering, University of Leeds*

[*]E-mail: j.smith252@leeds.ac.uk

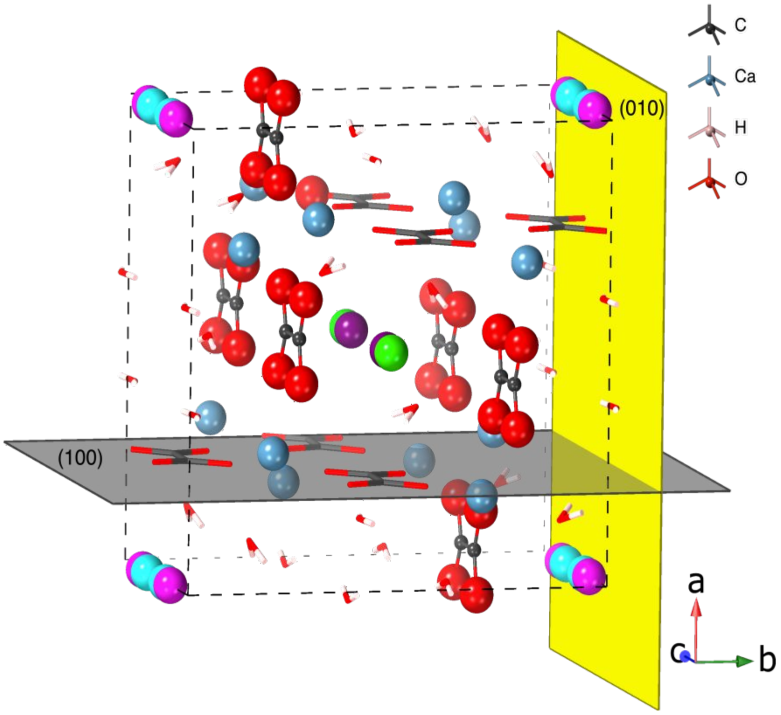

Supplementary Figure 1. Geometry optimised COD unit cells, showing alternative oxalate positions; Ox1 (stick) parallel to (100) plane, and Ox2 (spheres) parallel to (010) plane. Potential zeolitic water positions are indicated by purple, green,

turquoise and pink spheres. The purple spheres represent the lowest energy position.

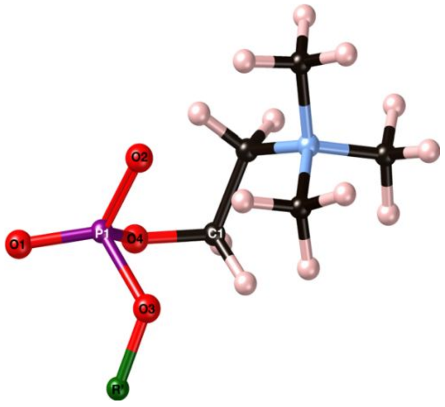

R'	Atomic Charges				
R'	O1/ |e|	O2/ |e|	O3/ |e|	P1/ |e|	C1/ |e|
A	-1.06	-1.10	-0.77	2.35	-0.26
B	-1.07	-1.11	-0.79	2.37	-0.25
C	-1.07	-1.11	-0.79	2.37	-0.26
D	-1.07	-1.10	-0.79	2.37	-0.25
E	-1.10	-1.08	-1.06	2.30	-0.25
F	-1.08	-1.10	-1.05	2.36	-0.26

	Bond Lengths				
R'	P1-O1/ Å	P1-O2/ Å	P1-O3/ Å	P1-O4/ Å	C1-O4/ Å
A	1.479	1.516	1.663	1.669	1.411
B	1.480	1.509	1.656	1.691	1.409
C	1.479	1.511	1.653	1.689	1.409
D	1.480	1.509	1.656	1.691	1.409
E	1.510	1.505	1.693	1.700	1.475
F	1.480	1.510	1.662	1.688	1.408

	Mulliken Populations				
R'	P1-O1/ |e|	P1-O2/ |e|	P1-O3/ |e|	P1-O4/ |e|	C1-O4/ |e|
A	0.79	0.69	0.39	0.37	0.53
B	0.79	0.68	0.4	0.35	0.54
C	0.79	0.69	0.4	0.35	0.54
D	0.79	0.69	0.39	0.35	0.54
E	0.75	0.7	0.36	0.33	0.47
F	0.79	0.69	0.35	0.36	0.54

Supplementary Figure 2. LHS: Chemical structure of the phosphocholine model with R’ signifying the terminating groups in Figure 5. RHS: Table showing the atomic charges, bond lengths and Mulliken Populations for each terminating group. The atoms correspond to labels on the model.

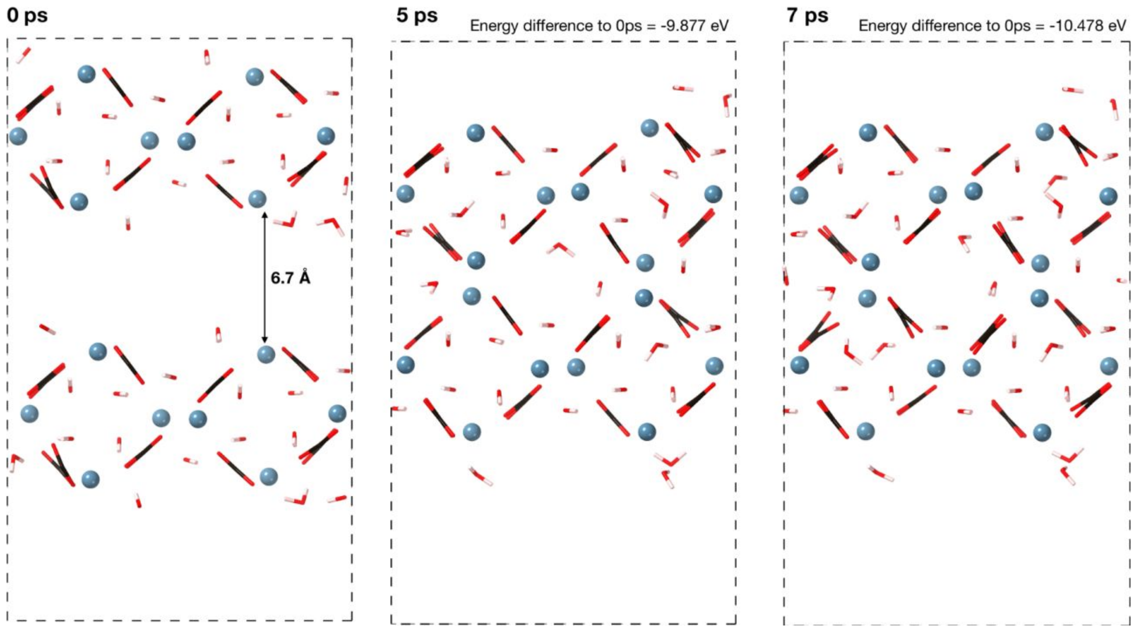

Supplementary Figure 3. AIMD control simulation of two COD (110) surfaces from 0 to 7 ps.
